# Spatial distribution of electrical reconnection after pulmonary vein isolation in patients with recurrent paroxysmal atrial fibrillation

**DOI:** 10.1007/s12471-016-0842-6

**Published:** 2016-05-24

**Authors:** L. M. Rademakers, I. Romero, T. A. Simmers, P. H. van der Voort, A M. Meijer, L. R. Dekker

**Affiliations:** Department of Cardiology, Catharina hospital, Eindhoven, The Netherlands; Philips Healthcare, Best, The Netherlands

**Keywords:** Atrial fibrillation, Pulmonary vein isolation, Reconnection

## Abstract

**Introduction:**

Recurrence of atrial fibrillation after pulmonary vein isolation (PVI) occurs frequently and may be associated with electrical reconnection of the pulmonary veins (PV). We investigated spatial distribution of electrical reconnection during re-do procedures in patients with paroxysmal atrial fibrillation who had previous successful acute electrical PVI with either single irrigated tip, antral ablation (s-RF; *n* = 38) or multi-electrode, duty-cycled ablation (PVAC; *n* = 48).

**Methods and Results:**

EP navigator, mapping and irrigated tip ablation catheters were used in all re-do procedures. Sites of reconnection were assessed in a 12-segment model. Baseline clinical and demographic characteristics were similar in both groups. The number of PVs reconnected per patient was similar in both groups (2.9 ± 0.9 and 3.2 ± 0.7 (*p* = 0.193), s‑RF and PVAC, respectively), and each PV was equally affected. However, the inferior quadrant of the right lower PV was significantly more vulnerable to reconnection after previous PVAC ablation, whereas the superior quadrant of the right upper PV showed significantly more reconnection in the s‑RF group.

**Conclusion:**

The overall number of PVs reconnected was equally high in both groups, and each PV was affected equally. However, there were significant differences in the spatial distribution of electrical reconnection. Better understanding of predilection sites for reconnection might help to improve the long-term success rate of PVI.

## Introduction

Pulmonary vein isolation (PVI) has emerged as an important therapeutic option in patients with drug-refractory atrial fibrillation (AF). Various techniques have been applied to electrically disconnect the pulmonary veins (PVs) from the adjacent atrium (reviewed in Dewire et al. [1]). Although these techniques are often successful in achieving acute electrical isolation of the PVs, in the long term success is still low with up to 30 % of patients with paroxysmal AF suffering recurrence of AF within 2 years after PVI [2]. This recurrence may be related to recovery of electrical conduction in a previously isolated pulmonary vein (PV). Reconnection was found in 61–97 % of previously isolated PVs in patients undergoing additional PVI after AF recurrence [3–5]. In addition, non-PV foci [3, 5–7] and changes in the atrial substrate due to coexistent diseases such as arterial hypertension or diabetes may cause recurrent AF [8].

In this prospective study, we investigated the number of PV reconnections and its spatial distribution after two different techniques used for PVI for the index procedure, i. e. single irrigated tip, antral ablation (s-RF) and multi-electrode, duty-cycled ablation (PVAC). Better understanding of the spatial distribution of reconnection might help to improve the long-term success rates of these techniques after the first procedure.

## Methods

### Population

This study was approved by the Medical Ethics Committee of our institution and all patients gave written informed consent.

Data were collected between November 2011 and June 2014. Eighty-six consecutive patients previously successfully treated with PVI for paroxysmal AF and accepted for a second procedure due to recurrent symptomatic paroxysmal AF were eligible for inclusion in this study. A team consisting of four experienced electrophysiologists performed all PV isolations (both initial and second procedures). Patients who had an unsuccessful first procedure, i. e. no acute and complete isolation of all PVs, or who had more than one previous PVI, were not eligible for this study. In addition, patients with a left common PV, with five PVs or with atrial tachyarrhythmias other than AF were also excluded from this study.

### Initial procedure

The initial procedure was performed using one of two different techniques, both routinely performed. In one group of patients (*n* = 38), a single tip, 4 mm irrigated RF ablation catheter (s-RF) was used in combination with a CARTO (Biosense Webster Inc., CA, USA) or EnSite NavX (St. Jude Medical Inc., MN, USA) navigation system. In this population point-by-point wide antral circumferential ablation was performed, with an irrigated single tip ablation catheter without contact force feedback (information about adequacy of tip-to-tissue contact, aimed to optimise energy delivery), since this option was not available at the time of the index procedure. In the other group (*n* = 48), a multi-electrode, duty-cycled (technology combining uni- and bi-polar ablation) RF catheter (PVAC, Medtronic Inc., MN, USA) was used with an EP navigator (Philips Medical Systems, Eindhoven, the Netherlands) for imaging support. In patients treated with the latter catheter type, a non-steerable transseptal sheath was used. For both groups, disappearance of PV signals and exit block during pacing from the PVs were used to assess procedural acute success after a waiting time of 30 minutes. Adenosine or other additional means to assess dormant reconnections were not used [9].

### Second procedure

**Fig. 1:**
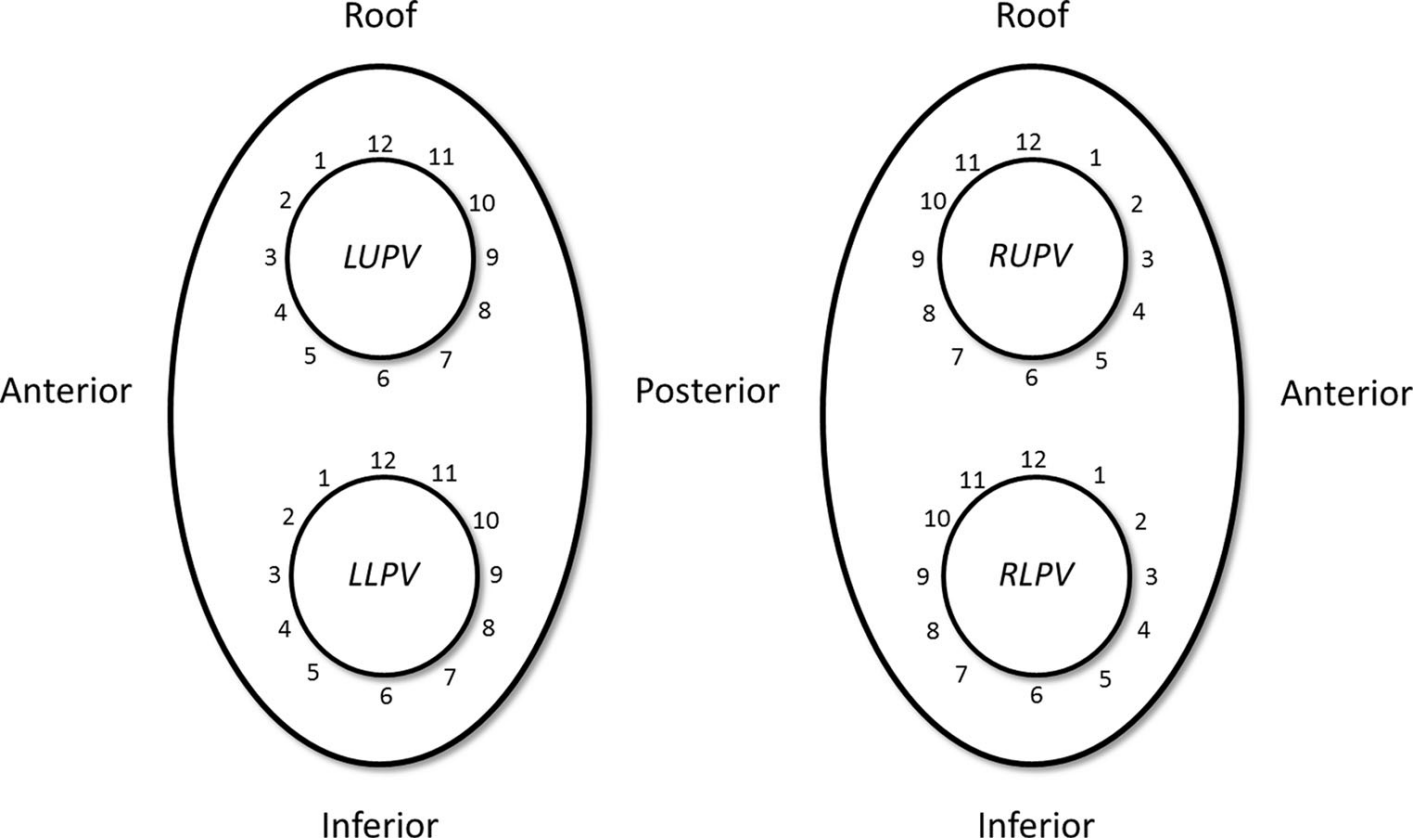
Diagram of pulmonary veins. Schematic representation of the four pulmonary veins in PA view (*LUPV* left upper pulmonary vein, *LLPV* left lower pulmonary vein, *RUPV* right upper pulmonary vein, *RLPV* right lower pulmonary vein)

All patients were treated according to the same routine procedure for recurrence of paroxysmal AF, independent of the technique used in the initial procedure. In this second procedure only X-ray was used for imaging. In patients treated with warfarin, oral anticoagulation was not interrupted, whereas novel oral anticoagulants (NOACs) were stopped 24 hours before the procedure. During the intervention, all patients were heparinised to achieve an activated clotting time >300 seconds, after double transseptal puncture. All PVs were assessed for the presence and the exact location of electrical reconnection using a circular mapping catheter (either a Lasso™ [Biosense Webster Inc., CA, USA] or a Reflection™ [St. Jude Medical Inc., MN, USA] catheter). A reconnection site was defined as the location in the previous ablation line that was associated with the site of earliest activation as assessed by the circular mapping catheter, which was placed just distal from the ablation line. In case of multiple sites of reconnection, the site of earliest activation was always targeted first. The location of reconnection was expressed by projecting a virtual clock around each PV ostium with the left PVs and right PVs in RAO and LAO projections, respectively. The operator determined, based on X‑ray imaging, the location of reconnection by linking it to one of the 12 hours in the virtual clock. In the left PVs, 3 and 9 o’clock corresponded to the centre of the anterior and posterior quadrant, respectively, in contrast to the right PVs where 3 o’clock and 9 o’clock corresponded to the centre of the posterior and anterior quadrant, respectively. See Fig. 1 for schematic representation. Sites of reconnection were ablated using a single tip irrigated RF ablation catheter (CELSIUS Thermo-Cool, Biosense Webster Inc., CA, USA or Quickflex, St. Jude Medical Inc., MN, USA). Each ablation was applied for 60 seconds at a maximum of 30 Watts and was repeated if necessary. After successful local ablation, the PV was checked for additional reconnections and treated in the same way as described above, if necessary until complete PVI was achieved.

### Statistical analysis

Continuous data are presented as mean and standard deviation and discrete variables as counts and percentages, unless otherwise stated. No missing data imputation was performed. Nonparametric tests, logistic regression analysis and fixed effect analysis were used for the comparison within and between the two groups. A two-sided *p* value of <0.05 was considered statistically significant.

## Results

Tab. 1 displays the baseline clinical and demographic patient characteristics. Eighty-six patients (age 62 ± 9, 60 men) underwent a second electrophysiological study and PVI. Patients had 1.7 ± 0.7 failed antiarrhythmic drugs before the first procedure. Time interval from initial to second PVI was 16.5 ± 10.8 months and 10.9 ± 7.5 months for s‑RF and PVAC, respectively (*p* = 0.008). All re-do procedures were successful, i. e. in all patients complete acute electrical re-isolation was achieved, and there were no major procedural complications, such as cerebrovascular accidents, pericardial effusion or groin haematoma requiring surgical exploration.

**Tab. 1:** Baseline clinical and demographic characteristics

Parameter	s-RF ablation	PVAC ablation	*p* value
Age at repeat PVI, years	62.9 ± 9.7	60.2 ± 8.8	0.215
*Sex, n (%)*			*0.155*
Male	30 (79)	30 (63)	
Female	8 (21)	18 (37)	
AF duration before index PVI, years	5.5 ± 5.2	5.4 ± 4.4	0.935
Time from initial to repeat PVI, months	16.5 ± 10.8	10.9 ± 7.5	0.008
*History, n (%)*			
Hypertension	21 (55)	20 (42)	0.210
Diabetes	1 (3)	2 (4)	0.588
Congestive heart failure	0 (0)	0 (0)	1.0
Stroke	2 (5)	3 (6)	0.611
Peripheral artery disease	4 (11)	4 (8)	0.728
Coronary artery disease	4 (11)	6 (13)	0.526
Moderate or severe valvular disease	0 (0)	0 (0)	1.0
No. of failed antiarrhythmic drugs (before initial procedure)	1.8 ± 0.7	1.6 ± 0.8	0.354
*Antiarrhythmic drugs (before initial procedure), n (%)*			
Beta blocker	15 (31)	29 (60)	0.007
Sotalol	11 (29)	29 (60)	0.004
Amiodarone	6 (16)	9 (19)	0.719
Flecainide	28 (74)	23 (48)	0.016
Propafenone	0 (0)	3 (6)	0.082
*Echocardiographic parameters*			
LA dimension (mm)	40.6 ± 5.9	37.6 ± 7.7	0.112
LA volume (ml/m^2^ BSA)	33.0 ± 9.6	28.0 ± 9.3	0.075

*AF* atrial fibrillation,* BSA* body surface area, *LA* left atrial, *PVAC ablation* multi-electrode, duty-cycled ablation, *PVI* pulmonary vein isolation, *s-RF ablation* single irrigated tip radiofrequency ablation

### Characteristics of electrical reconnection

All patients had reconnection of at least 1 PV, with 2.9 ± 0.9 and 3.2 ± 0.7 reconnected PVs for patients with previous s‑RF and PVAC procedures, respectively (*p* = 0.193). In each reconnected PV, there were 2.7 ± 1.6 and 2.9 ± 1.7 gaps of electrical connection found (s-RF and PVAC group, respectively [*p* = 0.779]) (Tab. 2).

**Tab. 2:** Procedural characteristics repeat pulmonary vein isolation

Parameter	s-RF ablation	PVAC ablation	*p* value (s-RF vs PVAC)
*No. of reconnected PVs, n (%)*			*0.193*
0	0 (0)	0 (0)	
1	2 (5)	1 (2)	
2	12 (32)	7 (15)	
3	11 (29)	22 (46)	
4	13 (34)	18 (37)	
*Reconnected PVs, n (%)*			*0.992*
LUPV	25 (71)	35 (80)	
LLPV	25 (71)	37 (84)	
RUPV	32 (84)	36 (75)	
RLPV	25 (66)	40 (83)	
*No. of reconnections per PV*			*0.779*
LUPV	2.9 ± 1.7	2.9 ± 1.6	
LLPV	2.4 ± 1.6	2.5 ± 1.3	
RUPV	2.8 ± 1.8	2.3 ± 1.1	
RLPV	2.8 ± 1.4	3.8 ± 2.1*	
*Duration of RF-energy application per gap, sec*			*0.892*
LUPV	76 ± 45	77 ± 48	
LLPV	70 ± 22	78 ± 45	
RUPV	75 ± 40	73 ± 39	
RLPV	66 ± 29	75 ± 42	
Procedure time, min	96.1 ± 27.5	97.5 ± 24.7	0.741
Fluoroscopy time, min	23.1 ± 10.9	28.0 ± 17.5	0.148
PV isolation, *n* (%)	38 (100)	48 (100)	1.0

*LUPV* left upper pulmonary vein, *LLPV* left lower pulmonary vein, *PVAC ablation* multi-electrode, duty-cycled ablation, *PV* pulmonary vein, *RUPV* right upper pulmonary vein, *RLPV* right lower pulmonary vein, *s-RF ablation* single irrigated tip radiofrequency ablation

* RLPV vs RUPV and LLPV, *p* = 0.001 and *p* = 0.004, respectively

**Fig. 2:**
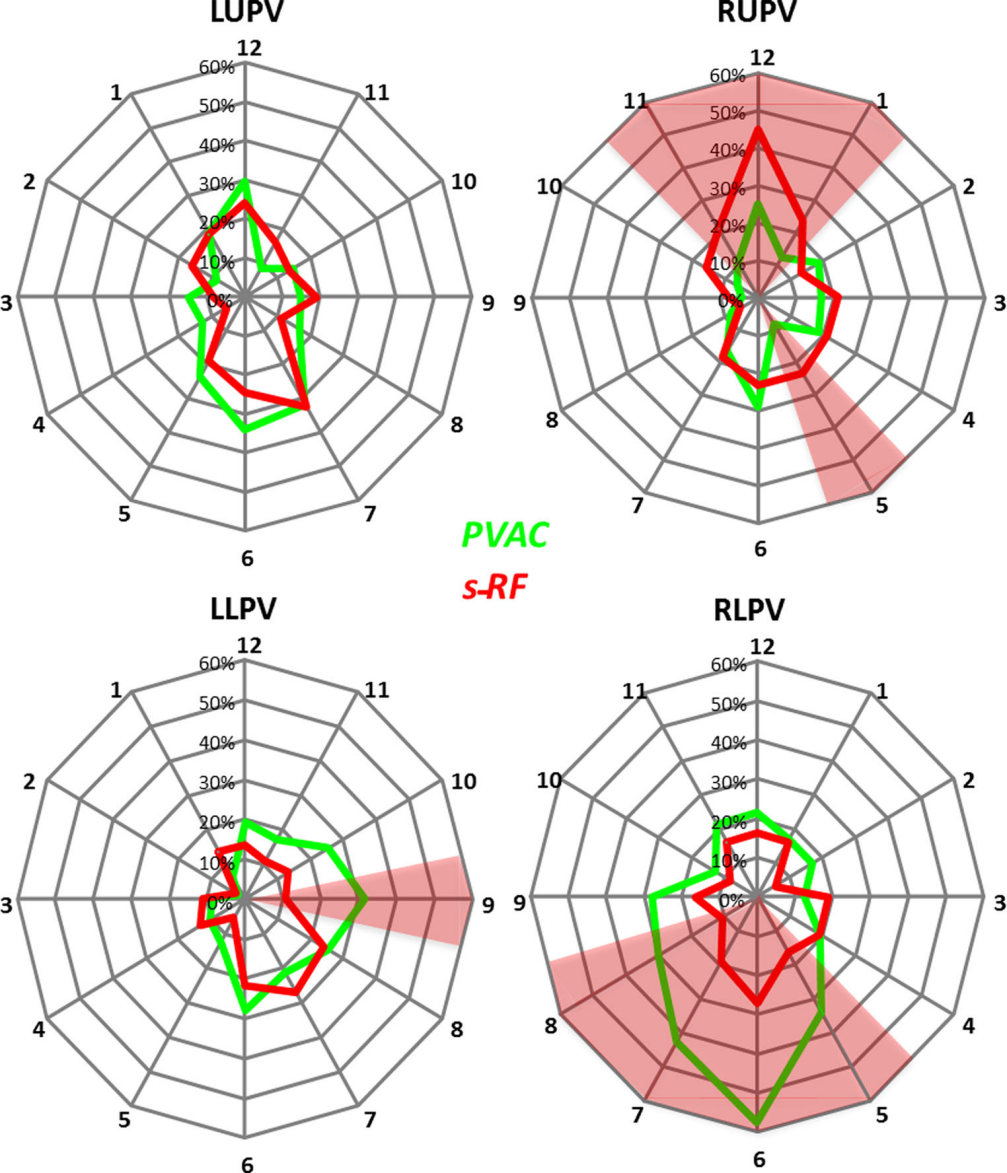
Spatial distribution of reconnection, in 12-segment model. Number of patients (as percentage) with electrical reconnection, expressed per segment in each of the four pulmonary veins. Figure in PA view. Red shaded areas indicate zones with statistically significant difference (*p* < 0.05) between the two techniques. *Green and red lines* represent PVAC and s‑RF, respectively (*LUPV* left upper pulmonary vein, *LLPV* left lower pulmonary vein, *RUPV* right upper pulmonary vein, *RLPV* right lower pulmonary vein)

There were predilection sites of electrical reconnection depending on the initial ablation technique. Fig. 2 displays the spatial distribution of electrical reconnection in each of the four PVs expressed in the 12-segment model. The red shaded area indicates the range with statistical difference (*p* < 0.05) when comparing the two techniques. The figure demonstrates that after previous s‑RF ablation reconnection is found more frequently superior in the right upper PV whereas in patients who had a previous PVAC ablation, reconnection was more often located inferior in the right lower PV. The figure also shows two additional small areas (1 segment each) in the right upper and left lower pulmonary vein in which there was a statistical difference between both techniques.

### Characteristics of RF application

The duration of RF-energy application per gap needed to achieve electrical re-isolation was 72 ± 36 and 75 ± 44 seconds for patients with previous s‑RF and PVAC ablation, respectively (*p* = 0.892). In both groups, the duration of RF-energy application for re-isolation was equally distributed between the PVs (Tab. 2) and there were no spatial differences with respect to the 12-segment model.

## Discussion

This is the first study comparing reconnection patterns of two commonly used ablation techniques and its main findings are: (1) reconnection occurred in all studied patients and, on average, 3 out of 4 PVs were reconnected; (2) although reconnection occurred equally among PVs and irrespective of the initial ablation technique, the superior region of the right upper PV is a predilection site for reconnection after single point RF ablation as compared with the inferior region of the right lower PV after a previous PVAC procedure; (3) the duration of RF-energy application necessary to re-isolate a site of reconnection is similar in both groups.

PVI is the cornerstone in ablation therapy for paroxysmal AF; however, electrical reconnection is a common finding after PVI. The current study as well as previous reports indicate that reconnection is associated with clinical AF recurrence [4, 5, 10]. Verma et al. [11] correlated long-term cure with PV isolation and showed that the majority of patients who were free from AF after PVI had no recurrent PV conduction, whereas all patients with recurrent AF had reconnection. Cappato et al. [12] investigated the correlation between acute achievement and chronic maintenance of electrical conduction block after classic point-by-point PVI for paroxysmal or persistent AF. Late PV conduction recurrence was a frequent finding: approximately 80 % of all ablated PVs showed recurrent conduction after 4.5 months, irrespective of the symptoms [12].

Non-PV foci [3, 5–7] and changes in the atrial substrate due to coexistent diseases such as arterial hypertension or diabetes may also explain some of the recurrences [8].

Rajappan et al. [13] investigated the anatomical sites of reconnection after previous successful PVI using single point RF ablation. In agreement with our data, reconnection in the right upper PV was observed predominantly superiorly, at the junction of the roof of the left atrium. These findings might be explained by a significant variation in the transmural myocardial thickness of the venoatrial junction, which is greatest at the intervenous ridge [14]. In addition, in this region adequate tissue contact may be difficult to achieve due to a curved routing of the ablation catheter.

In patients with recurrent AF who underwent a second procedure after PVI by PVAC, the reconnection rate was 73 % of all previously isolated PVs [15]. Balt et al. [16] reported that in almost all patients (98 %) with recurrent AF after previous PVAC ablation at least one PV was reconnected, and all PVs were equally likely to show reconnection. Other studies demonstrated that superior veins were more often affected as compared with the inferior ones [9, 17–19]. In our study, as well as in a study by Brunelli et al. [20], the highest rate of reconnection was observed for the inferior quadrant of the right lower PV, most likely due to difficulties in appropriately engaging this vein with the PVAC and early branching of this vein [21]. Optimal electrode-tissue contact with all electrode pairs may be more difficult to achieve due to the circular design of the catheter.

In order to reduce PV reconnections, longer waiting periods and additional pharmacological testing, e. g. using adenosine, revealing ‘dormant’ PV conduction, have been proposed [20]. When isolation was assessed 30 minutes after electrical disconnection using an irrigated catheter, up to 33 % of the PVs recovered conduction, and reconnection rates further increased at 60 minutes to a maximum of 50 % in up to 93 % of the patients [13, 22, 23].

A limitation of this study is that initial procedures were not randomised between the two techniques. However, patient baseline characteristics are comparable. Moreover, it is not likely that minor differences between patients might affect the results, since this is not a study on clinical results such as absence of symptoms. Exact information about the time interval between documented onset of AF recurrences and the second procedure is not available, but based on planning protocols, none of the re-do procedures took place within 4 months after the first procedure. It is generally assumed that the amount (and location) of reconnection of the PVs will not change beyond this 4‑month interval. A limitation is that this was a single-centre study, although our centre is a referral centre treating a high volume of patients. All PVAC procedures were performed using the first-generation, platinum electrode catheter with non-steerable sheaths, which may have hampered optimal tissue contact in the right lower PV. However, in the index procedure all patients had complete isolation of the right lower PV. Another limitation is that this study was carried out in a specific cohort of patients with a relatively non-complex arrhythmic substrate; PV reconnection characteristics may be different in patients with a more complex atrial substrate. Furthermore, although an experienced team of cardiologists equally participated in performing the index PV isolation following a standardised method, it cannot be excluded that predilection sites of reconnection are an operator-dependent effect.

## Conclusions

In all patients with recurrent AF there was electrical reconnection of at least one PV. Reconnection is more frequently found in the superior region of the right upper pulmonary vein after previous single point RF ablation whereas the inferior region of the right pulmonary vein is affected after previous PVAC ablation.

The number of reconnected veins is equally high for single tip RF and PVAC procedures (average of three PVs reconnected). For both index techniques areas with a high chance of reconnection are identified. It seems likely that these patterns are caused by the interaction between mechanical properties of the catheter and local anatomy. Better understanding of these patterns may help in designing better catheters and ablation protocols. Moreover, knowledge of these patterns may be of help for operators in performing better during the first procedure and reduce recurrence.
